# High Soluble Endoglin Levels Affect Aortic Vascular Function during Mice Aging

**DOI:** 10.3390/jcdd8120173

**Published:** 2021-12-04

**Authors:** Iveta Nejmanová, Barbora Vitverová, Samira Eissazadeh, Katarina Tripská, Ivone Cristina Igreja Sa, Radomír Hyšpler, Ivana Němečkova, Miguel Pericacho, Petr Nachtigal

**Affiliations:** 1 Department of Biological and Medical Sciences, Faculty of Pharmacy in Hradec Kralove, Charles University, Heyrovskeho 1203, 500 05 Hradec Kralove, Czech Republic; ivetanajmanova@seznam.cz (I.N.); vitverob@faf.cuni.cz (B.V.); eissazas@faf.cuni.cz (S.E.); tripskak@faf.cuni.cz (K.T.); igrejasi@faf.cuni.cz (I.C.I.S.); guncovai@faf.cuni.cz (I.N.); 2 Centrum for Research and Development, University Hospital, 500 05 Hradec Kralove, Czech Republic; RHyspler@lfhk.cuni.cz; 3 Renal and Cardiovascular Research Unit, Department of Physiology and Pharmacology, Biomedical Research Institute of Salamanca (IBSAL), University of Salamanca, 37007 Salamanca, Spain; pericacho@usal.es

**Keywords:** soluble endoglin, endoglin signaling, vascular function, mice

## Abstract

Endoglin is a 180 kDa transmembrane glycoprotein that was demonstrated to be present in two different endoglin forms, namely membrane endoglin (Eng) and soluble endoglin (sEng). Increased sEng levels in the circulation have been detected in atherosclerosis, arterial hypertension, and type II diabetes mellitus. Moreover, sEng was shown to aggravate endothelial dysfunction when combined with a high-fat diet, suggesting it might be a risk factor for the development of endothelial dysfunction in combination with other risk factors. Therefore, this study hypothesized that high sEng levels exposure for 12 months combined with aging (an essential risk factor of atherosclerosis development) would aggravate vascular function in mouse aorta. Male transgenic mice with high levels of human sEng in plasma (Sol-Eng^+^) and their age-matched male transgenic littermates that do not develop high soluble endoglin (Control) on a chow diet were used. The aging process was initiated to contribute to endothelial dysfunction/atherosclerosis development, and it lasted 12 months. Wire myograph analysis showed impairment contractility in the Sol-Eng^+^ group when compared to the control group after KCl and PGF_2__α_ administration. Endothelium-dependent responsiveness to Ach was not significantly different between these groups. Western blot analysis revealed significantly decreased protein expression of Eng, p-eNOS, and ID1 expression in the Sol-Eng^+^ group compared to the control group suggesting reduced Eng signaling. In conclusion, we demonstrated for the first time that long-term exposure to high levels of sEng during aging results in alteration of vasoconstriction properties of the aorta, reduced eNOS phosphorylation, decreased Eng expression, and altered Eng signaling. These findings suggest that sEng can be considered a risk factor for the development of vascular dysfunction during aging and a potential therapeutical target for pharmacological intervention.

## 1. Introduction

Endoglin is a 180 kDa transmembrane glycoprotein considered a coreceptor for ligands of the Transforming Growth Factor β (TGF-β) superfamily. There are two different endoglin forms, namely membrane endoglin (Eng) and soluble endoglin (sEng). The membrane form is expressed mainly by endothelial cells, vascular smooth muscle cells [1], fibroblasts [2], hepatic stellate cells [3], activated monocytes, and macrophages [4]. Eng associates with TGF-β receptors I (ALK-1, ALK-5) and II (TGF-βRII), enabling the binding of TGF-β1, TGF-β3, or bone morphogenic proteins (BMPs), thus regulating whether endothelial cells are activated or quiescent [5].

Vascular endothelium, the inner metabolically active part of blood vessels, regulates vascular tone by various signaling pathways [6]. One of the major mediators responsible for the regulation of vascular reactivity is nitric oxide (NO) [7]. This signaling molecule is an integral part of l-arginine/endothelial NO synthase (eNOS) cascade and is essential for maintaining physiological conditions of the cardiovascular system [8]. Indeed, lack of NO is associated with impaired vascular homeostasis; a state described as endothelial dysfunction [9].

Additionally, several studies showed the positive relationship between Eng, eNOS and proper function of vascular endothelium, where the decrease of endothelial Eng expression resulted in reduced eNOS expression, increased endothelial permeability, and destabilization of endothelial barrier, which are the hallmarks of endothelial dysfunction [10].

Soluble endoglin is the N-terminal cleavage product of the extracellular domain of Eng formed by the activity of matrix metalloproteinases (MMP14 and MMP12) that is released into the circulation or cell culture medium in various pathological conditions [11]. Increased sEng levels in the circulation have been detected in endothelium-related pathologies such as atherosclerosis [12], arterial hypertension, and type II diabetes mellitus [13]. Moreover, sEng is not only a biomarker of cardiovascular and metabolic disorders. Recently, it was demonstrated that sEng regulates bone morphogenetic protein-4 (BMP4) levels, resulting in the induction of arterial hypertension in mice and cardiac hypertrophy/heart failure, which suggests BMP4 as a downstream mediator of sEng [14,15]. Our previous study showed no effect of short exposure of high sEng levels (up to 5 months) on endothelial function in mice [16]. Interestingly, a combination of high sEng levels and hypercholesterolemia (induced by a high-fat diet) affected endothelial function, resulting in the development of endothelial dysfunction [12,17]. All these data suggest the possible harmful effect of sEng on vascular function.

It is well known that mouse models are considered one of the favorite mammalian models in the field of aging [18]. Aging research provides understanding of correlative relationships between age-associated phenotypes in humans and mice [19]. Notably, mouse models represent a wide range of disease-associated phenotypes, including cardiovascular disease, comparable to the features of natural aging in humans [20]. Generally, 10–14-month-old mice are considered as middle-aged, reflecting equivalent to 38–47 human years [21]. Even though the outcomes from animal models must be taken with caution we used transgenic mice exposed to high sEng levels for 12 months in order to elucidate whether “aging” combined with high sEng levels will affect vascular function.

We hypothesized that high sEng levels exposure for 12 months combined with aging (an essential risk factor of atherosclerosis development) would aggravate vascular function in mouse aorta.

## 2. Materials and Methods

### 2.1. Animals and Experimental Design

Transgenic mice overexpressing human sEng on the CBAxC57BL/6J background were generated at the Genetically Modified Organisms Generation Unit (University of Salamanca, Spain), as previously described [22]. Male transgenic mice with high levels of human sEng in plasma (Sol-Eng^+^, ≥1000 ng/mL) and their age-matched male transgenic littermates that do not develop high soluble endoglin (Control animals in this study) on chow diet were used. As mentioned above, the aging process was initiated to contribute to endothelial dysfunction/atherosclerosis development, and it lasted 12 months. The animals were kept in controlled ambient conditions in a temperature-controlled room with a 12-h light/dark cycle with constant humidity. They had free access to tap water and chow rodent diet ad libitum. At 12 months, the mice were euthanized under general anesthesia induced by a combination of xylazine (10 mg/kg, i.p.) and ketamine (100 mg/kg, i.p.). Blood and aorta samples were harvested for further analysis.

All experiments were carried out according to a directive of the European Union 86/609/EEC. The Ethical Committee approved all procedures for the protection of animals against cruelty at the Faculty of Pharmacy, Charles University (Permit Number 4937/2019-8). All efforts were made to minimize the suffering of the animals.

### 2.2. Biochemical Analysis

The total concentration of plasma cholesterol and triglycerides were measured enzymatically by conventional diagnostic kits (Lachema, Brno, Czech Republic) and spectrophotometric analysis (cholesterol at 510 nm, triglycerides at 540 nm, Ultrospec III Spectrophotometer, Pharmacia LKB Biotechnology, Uppsala, Sweden).

### 2.3. Histological Analysis

The aortas, attached to the top half of the heart, were removed and then immersed in OCT (Optimal Cutting Temperature) embedding medium (Leica, Czech Republic), snap-frozen in liquid nitrogen, and stored at “80 C prior to histological analysis. Serial cross-sections (thickness 7 mm) of the thoracic part of the aorta were cut and stained with Hematoxylin for the histological evaluation.

### 2.4. ELISA

The plasma levels of human and mouse sEng were determined using a Human Endoglin/CD105 and a Mouse Endoglin/CD105 Quantikine ELISA Kit (R&D Systems, Minneapolis, MN, USA) according to the manufacturer’s instructions. Blood samples were obtained from the vena cava inferior.

### 2.5. Functional Analysis of Vascular Reactivity Ex Vivo

Aortic rings underwent cleaning, mounting, and measuring processes as previously described by Vitverova et al. [12].

### 2.6. Western Blot Analysis

The procedure was performed as previously reported by Nemeckova et al. [16]. Equal loading of proteins onto the gel was confirmed by immunodetection of mouse monoclonal Anti-GAPDH antibody. The bands were detected by ChemiDoc^TM^ MP Imaging System (Bio-Rad Laboratories, Hercules, CA, USA). Quantification of bands was performed by Image Lab software (Bio-Rad Laboratories, Hercules, CA, USA, version 6.0.1). Specific antibodies are listed in Table 1.

### 2.7. Statistical Analysis

The statistical analysis was performed by GraphPad Prism software version 9.0.0 (GraphPad Software, Inc., San Diego, CA, USA). All data are presented as a mean ± S.E.M.; direct group-group comparisons were carried out using a non-parametric Mann–Whitney test. *p* values of 0.05 or less were considered statistically significant.

## 3. Results

### 3.1. Lipid Spectrum, Human and Mouse sEng Levels in Plasma

**Figure 1:**
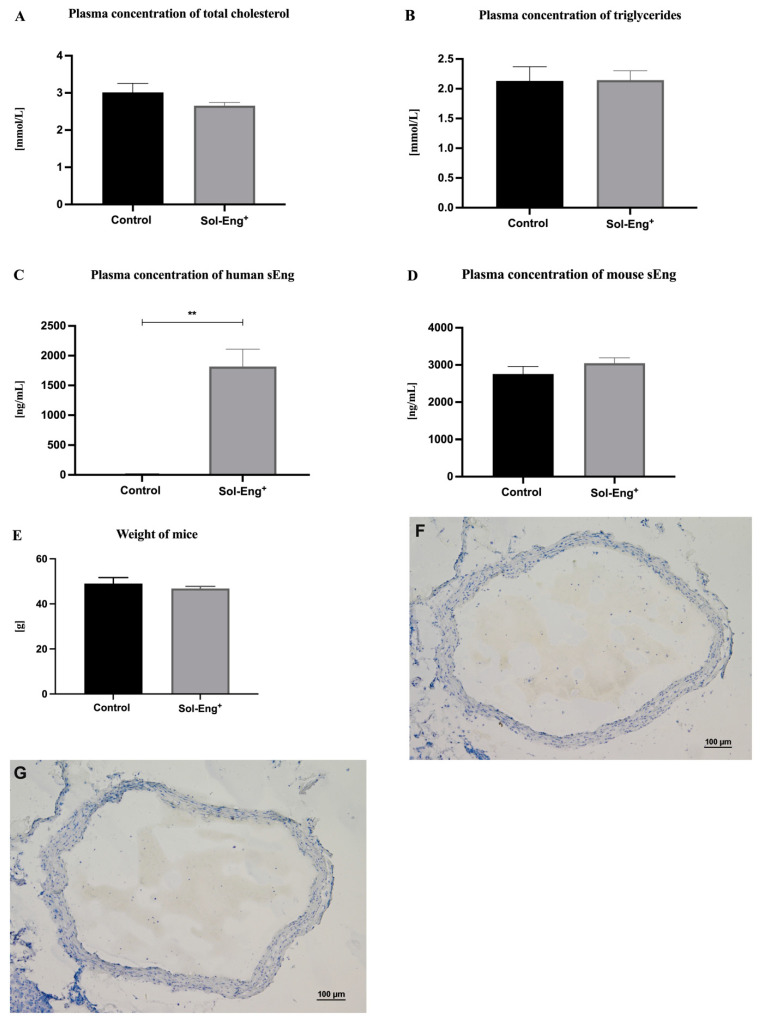
Biochemical and ELISA analyses of plasma. Plasma concentration of total cholesterol (**A**), triglycerides (**B**), human sEng (**C**), mouse sEng (**D**), and weight (**E**) in Sol-Eng^+^ mice and control group. Representative microphotographs of hematoxylin-stained aortas from control group (**F**) and Sol-Eng^+^ (**G**) mice. Data are shown as a mean ± S.E.M., Mann–Whitney test, ** *p* ≤ 0.01. *n* = 6 mice per group.

To define lipid profile and sEng levels, biochemical and ELISA analyses were performed. Plasma concentration of cholesterol levels (Figure 1A) and triglycerides (Figure 1B) did not significantly differ between the groups. As expected, the significantly higher levels of human sEng were detected in the plasma of Sol-Eng^+^ mice (1818.18 ± 291.59 ng/mL) compared to the control group (5.74 ± 11.76 ng/mL) (Figure 1C). Plasma levels of mouse sEng and mice weight were not significantly different between the groups (Figure 1D,E). Histological evaluation of thoracic aortas did not reveal any visible structural changes in any aortas from both groups (Figure 1F,G).

### 3.2. Impaired Vascular Contractility in Aortas of Sol-Eng^+^ Mice

To assess the impact of 12-month exposure of human sEng on aortic function, the parameters of vascular contractility and relaxation were evaluated.

**Figure 2:**
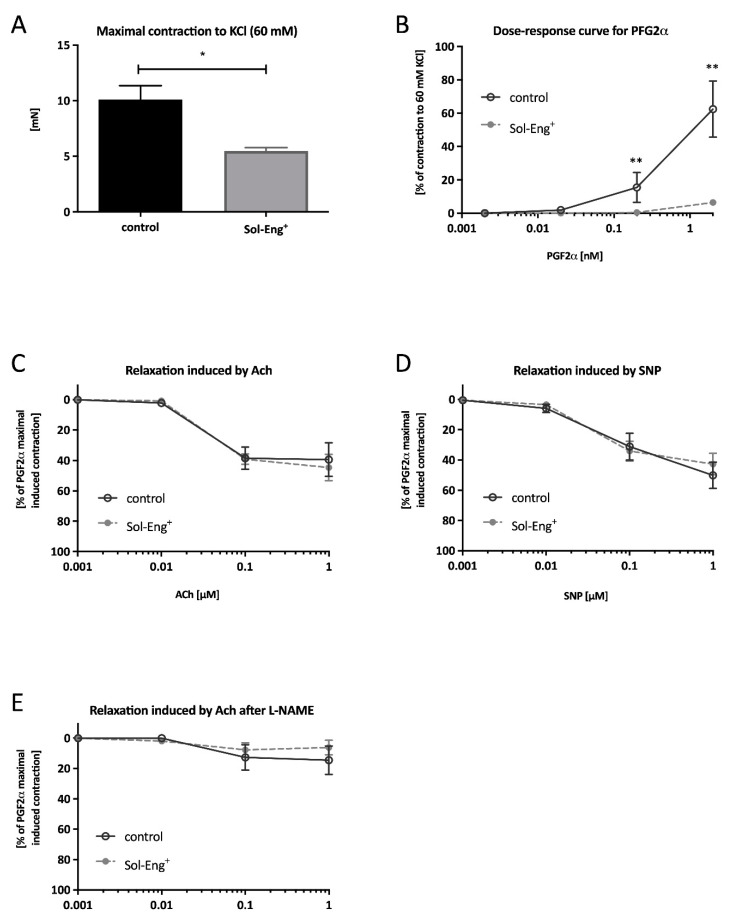
Functional analysis of vascular reactivity. A maximal contraction to KCl (60 mM) (**A**), dose–response curve to cumulative addition of PGF_2α_ (**B**), dose–response curve to cumulative addition of Ach in pre-constricted aortic segments by PGF_2α_ (**C**), dose–response curve to cumulative addition of SNP in pre-constricted aortic segments by PGF_2α_ (**D**), dose–response curve to cumulative addition of Ach in pre-constricted aortic segments by PGF_2α_ in the presence of L-NAME (**E**). Data are shown as a mean ± S.E.M., Mann–Whitney test, * *p* < 0.05, ** *p* ≤ 0.01. *n* = 6 mice per group.

KCl-induced vascular smooth muscle contraction response was significantly lower in Sol-Eng^+^ mice compared to the control group (10.11 ± 1.24 vs. 5.47 ± 0.32 mN), as shown in Figure 2A.

To check vasoconstriction capability of aortic segments (besides KCl stimulation), the cumulative PGF_2__α_ concentration in range 0.001–1 μM was directly added into the chambers. Impairment of contractility was observed in Sol-Eng^+^ group when compared to the control group at the point corresponding to concentration 0.1 μM (0.47 ± 0.34% vs. 15.50 ± 8.97%) and 1 μM (6.47 ± 1.14% vs. 62.50 ± 16.88%) (Figure 2B). Collectively, these data suggest impaired vasoconstriction and inability to maintain vascular tone in Sol-Eng^+^ mice.

To determine whether the ability of relaxation is also impaired, acetylcholine (Ach) and sodium nitroprusside (SNP) was used. Nevertheless, endothelium-dependent responsiveness to Ach was not significantly different between the groups (Figure 2C). However, the cumulative concentration line of SNP did not result in any significant differences in the level of relaxation between the groups (Figure 2D).

In addition, to reveal the possibility of auxiliary mechanism generating NO, relaxation mediated by Ach was also measured in the presence of N (ω)-nitro-l-arginine methyl ester (L-NAME), a specific inhibitor of eNOS. As presented in Figure 2E, no significant relaxation in response to Ach in the presence of L-NAME is observed in none of the groups, suggesting that the level of relaxation induced by Ach in Figure 2C is completely mediated by the relaxing effect of NO coming from the l-arginine/eNOS signaling cascade.

### 3.3. High sEng Levels Do Not Affect the Expression of Biomarkers of Endothelial Dysfunction

Biomarkers of endothelial dysfunction, namely vascular cell adhesion molecule 1 (VCAM-1) and intercellular adhesion molecule 1 (ICAM-1), were evaluated in the aorta.

**Figure 3:**
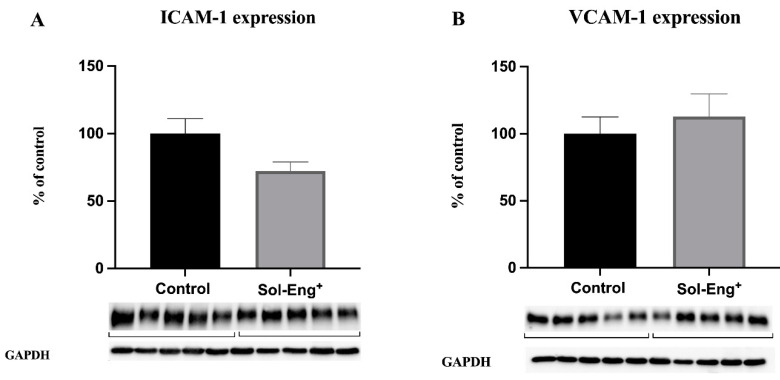
Expression of endothelial adhesion molecules in aortas of twelve-month-old male Sol-Eng^+^ and control mice. Expression of ICAM-1 (**A**) and VCAM-1 (**B**) in total protein extract from mice aortas. Densitometric quantification of immunoreactive bands (top panel: densitometric analysis, control = 100%) was recalculated to the GAPDH signal (bottom panel: representative immunoblots). Data are shown as a mean ± S.E.M, Mann–Whitney test. *n* = 5 mice per group.

The results of Western blot analysis showed no significant difference between Sol-Eng^+^ and the control group regarding the protein expression of these biomarkers (Figure 3A,B).

### 3.4. High sEng Levels Inhibit Membrane Endoglin Downstream Signaling

**Figure 4:**
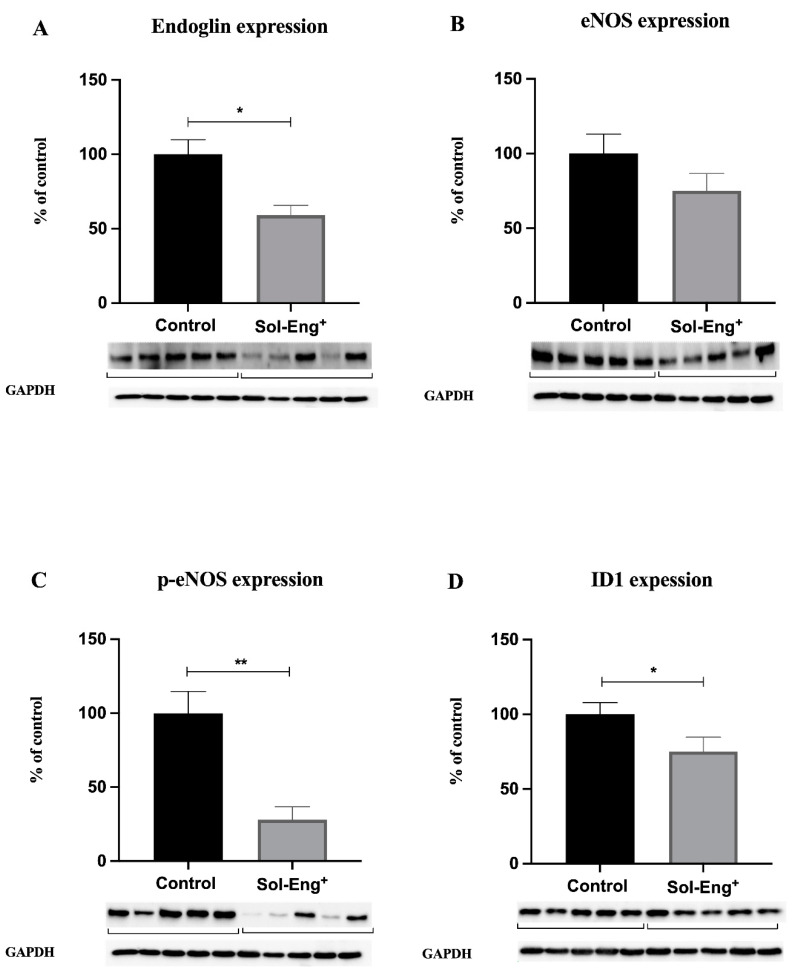
Expression of membrane endoglin and selected molecules belonging to its signaling pathway in aortas of twelve-month-old male Sol-Eng^+^ and control mice. Expression of membrane endoglin (**A**), eNOS (**B**), p-eNOS (**C**), and ID1 (**D**) in total protein extracts from mice aortas. Densitometric quantification of immunoreactive bands (top panel: densitometric analysis, control = 100%) was recalculated to the GAPDH signal (bottom panel: representative immunoblots). Data are shown as mean ± S.E.M, Mann–Whitney test, * *p* ≤ 0.05, ** *p* ≤ 0.01. *n* = 5 mice per group.

We aimed to evaluate the effects of high sEng levels with respect to endoglin-related signaling, including eNOS, p-eNOS, and Id1. Western blot analysis revealed significantly decreased protein expression of membrane endoglin (to 59%, Figure 4A) and p-eNOS (to 28%, Figure 4C) in the Sol-Eng^+^ group compared to the control group, suggesting altered endoglin signaling. On the other hand, as shown in Figure 4B, eNOS protein expression did not reach statistical significance between the groups. In addition, significantly decreased expression of ID1 was observed in the Sol-Eng^+^ group compared to the control group (to 75%, Figure 4D), suggesting reduced Eng signaling.

### 3.5. High Levels of sEng Affect the Expression of Markers Related to Vascular Contractility

**Figure 5:**
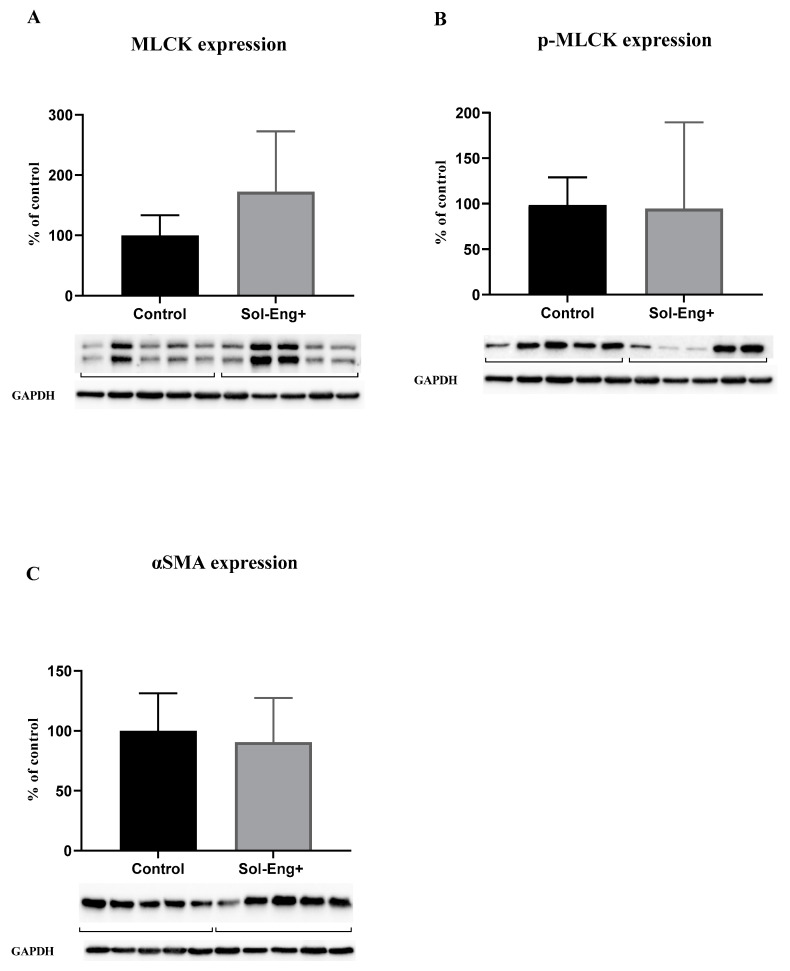
Expression of markers related to smooth muscle cell reactivity. Expression of MLCK (**A**), p-MLCK (**B**), and αSMA (**C**) in total protein extract from mice aortas. Densitometric quantification of immunoreactive bands (top panel: densitometric analysis, control = 100%) was recalculated to the GAPDH signal (bottom panel: representative immunoblots). Data are shown as mean ± S.E.M, Mann–Whitney test. *n* = 5 mice per group.

Myosin light chain kinase (MLCK) protein levels in the aorta were evaluated in order to reveal the potential impact of sEng on vascular contractility. To express the real activity and contribution of MLCK to vasoconstriction capability, we determined the expression of MLCK (Figure 5A) and its phosphorylated form (Figure 5B). As shown in Figure 5A,B, the Sol-Eng^+^ group has a similar expression of MLCK and p-MLCK as the control group.

Alpha smooth muscle actin (αSMA) expression was measured to detect possible changes in the smooth muscle cells layer (with respect to vascular motility and contraction) in aortic tunica media. However, we did not observe any significant effect of high sEng levels on αSMA expression between the groups (Figure 5B).

## 4. Discussion

Soluble endoglin represents an interesting biomarker of vascular pathology in both clinical and experimental studies [23]. More interestingly, several studies demonstrated potential harmful effects of sEng in endothelial function [12], liver metabolism [24], and development of NASH [25], suggesting that high levels of sEng might be considered as a risk factor of development of vascular alteration. Indeed, placenta-derived sEng from pregnant preeclamptic women is able to increase vascular permeability and induce arterial hypertension in vivo, suggesting that sEng plays an essential role in the pathogenesis of arterial hypertension, proteinuria, and glomerular endotheliosis [26]. It was also demonstrated that sEng treatment resulted in activation of NF-κB and IL-6, suggesting an activation of a proinflammatory phenotype in endothelial cells in vitro [27]. Transgenic mice with high levels of sEng develop preeclampsia symptoms, including high blood pressure and proteinuria [28].

Thus, we hypothesized that twelve months of exposure to high levels of sEng simultaneously with the aging of these mice will affect aortic function.

It was demonstrated that 6 months of exposure to high sEng levels did not affect the endothelial and vascular function of the aorta in our previous study [16]. Interestingly, once a high-fat diet was added for three months (to six months old mice with high sEng levels), it resulted in the induction of pro-inflammatory and pro-oxidative phenotype [17]. Furthermore, when the high-fat diet was added for six months (to three months old mice), it resulted in impaired KCl induced vasoconstriction, Ach-dependent vasodilation, and endothelial-independent relaxation induced by SNP. Additionally, Eng, pSmad2/3/Smad2/3, p-eNOS/eNOS signaling pathway was significantly reduced in Sol-Eng^+^ mice [12]. These data suggested that sEng may require another risk factor to induce endothelial dysfunction phenotype in the aorta.

Aging is considered a non-modifiable risk factor for the development of endothelial dysfunction and atherosclerosis [29]. In this study we used, 12-month-old transgenic mice with highs Eng levels, which should reflects 50-years-old humans [21].

It was well established that Sol-Eng^+^ mice are hypertensive [26]; however, the precise mechanism of arterial hypertension development is still unknown. In this study, the analysis of vasoconstriction properties of the aorta revealed reduced contractile capacity after KCl and PGF_2__α_ in Sol-Eng^+^ mice. Potassium chloride (KCl) is involved in the activation of voltage-operated Ca^2+^ channels resulting in elevation of Ca^2+^ concentration in the cytosol [30]. This stimulus leads to phosphorylation of the myosin light chain with subsequent contraction [31]. Prostaglandin F2 alpha (PGF_2α_) stimulates vasoconstriction through PGF_2α_ receptor localized in smooth muscle cells [32]. Even though the aorta is not a crucial vessel regulating blood pressure, we might speculate that the aorta of Sol-Eng^+^ mice that are hypertensive is already in a higher contractility state, therefore, cannot be contracted by KCl and PGF_2__α_. This suggests impaired vasoconstriction and inability to maintain vascular tone after long-term exposure to high sEng levels.

In addition, we aimed to evaluate the molecular background of the contractility alterations. Myosin light chain kinase (MLCK) has an essential role in the initiation of smooth muscle cell contraction [33]. Phosphorylation of MLCK (p-MLCK) leads to an increase in its activity [34]. However, Western blot analysis did not show a significant change in p-MLCK expression in Sol-Eng^+^ mice. Thus, the precise, complex mechanism of how sEng affects vascular contractility remains to be clarified.

Furthermore, we evaluated the Ach-dependent vasodilation in these mice. Ach stimulates muscarinic receptor subtype III in the endothelium [35]. This activation leads to a potent relaxation via releasing of NO [36,37]. The previous study showed no change in Ach-dependent vasodilation in younger mice exposed to high levels of sEng for six months [16]. However, once a high-fat diet was combined with high sEng levels, Ach-dependent vasodilation was altered, and the development of endothelial dysfunction was confirmed, suggesting that hypercholesterolemia combined with high sEng levels represent risk factors for the development of endothelial dysfunction [12]. In this study, Ach-dependent vasodilation was not significantly affected in 12 months old Sol-Eng^+^ mice, which might suggest that sEng requires another atherogenesis risk factor to be harmful to the endothelium.

On the other hand, it is interesting to mention that Ach-dependent vasodilation was very low in control animals in this study. This is in line with the paper mentioned above, where we found a very low level of Ach-dependent vasodilation in mice even without high sEng levels [12]. These data suggest that this mouse strain, which provides the molecular background for generating mice with high sEng levels, spontaneously develops the alteration of Ach-dependent vasodilation with age, regardless of the presence of high sEng levels or hypercholesterolemia.

It is of interest to emphasize that the development of endothelial dysfunction in the aorta is related to reduced Eng/eNOS expression in the aorta and decreased NO production in the ApoE^−/−/^LDLR^−/−^ hypercholesterolemic mouse model of endothelial dysfunction and atherogenesis [38]. In addition, it was shown that Eng/eNOS signaling/expression is reduced with atherogenesis progression and development of endothelial dysfunction in hypercholesterolemic mice [12,23]. Indeed, Eng stabilizes eNOS function and increases its expression [39,40]. It was proposed that Eng promoting eNOS expression and activity represents an important mechanism for proper endothelial function in the aorta [10]. In addition, the phosphorylation process of eNOS (p-eNOS) at the position of serine 1177 directly leads to an increase in NO production [41]. Inhibitor of DNA binding 1 (ID1) is important for normal response to injury [42]. Moreover, it is a downstream product of endoglin/Smad1/5/8 signaling [5]. In this study, downstream molecules of the Eng pathway, including eNOS/p-eNOS and ID1, were reduced, suggesting that sEng reduces Eng signaling in the aorta. These changes were not detected in younger six-months-old Sol-Eng^+^ mice [16], but they are in line with the paper of Vitverova et al., where high sEng levels combined with mild hypercholesterolemia resulted in alteration of NO production due to altered eNOS signaling in the aorta [12]. Therefore, we propose that the high levels of sEng combined with aging result in the same alteration of Eng/eNOS expression/signaling as sEng combination with hypercholesterolemia.

Interestingly, it was demonstrated that sEng treatment resulted in the activation of NF-κB and IL-6, suggesting an activation of a pro-inflammatory phenotype in endothelial cells [40]. Indeed, vascular cell adhesion molecule 1 (VCAM-1) and intercellular adhesion molecule 1 (ICAM-1) play an important role in the initiation of inflammation and endothelial dysfunction [43]. ICAM-1 has a major effect on leukocyte-endothelium interaction in the regulation of vascular permeability [44]. In addition, VCAM-1 is considered an endothelial cell activation/dysfunction marker, and it mediates leukocyte adhesion and trafficking [45,46]. However, the analysis of the protein expression of the VCAM-1 and ICAM-1 did not reveal significant changes in mice with high sEng levels, which suggests that sEng alone does not induce endothelial inflammation. Interestingly, as shown previously, combination of sEng and high-fat diet resulted in an increase of cell adhesion molecules [17], suggesting critical importance of atherogenic diet in the induction of endothelial inflammation.

In this study, the only difference between mice was the presence of high sEng levels without significant differences in cholesterol and triglyceride levels. Therefore, we may ask the question if sEng can affect the expression and/or signaling of membrane Eng.

**Figure 6:**
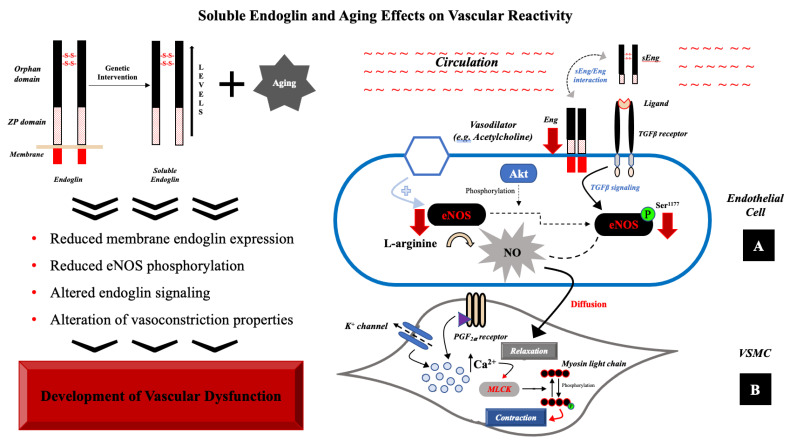
Role of sEng with aging in vascular dysfunction. (**A**) In endothelial cells, sEng and aging reduce Eng expression (red arrow) and signaling resulting in reduced expression of eNOS (red arrow) and its reduced phosphorylation rate (red arrow). (**B**) Vasoconstriction process, localized in VSMC, consists in phosphorylation of myosin light chain by an activated form of myosin light chain kinase (MLCK). Binding to PGF_2__α_ receptor or activation of K^+^ channel evoke changes in intracellular milieu, increasing calcium ion concentration. The presence of high levels of sEng and aging probably contribute to the alteration of vasoconstriction.

It has been postulated that sEng can antagonize the Eng function by occupying the Eng binding site [47,48]. sEng has been shown to not only inhibit the interaction between Eng and leukocyte integrins resulting in the inhibition of leukocytes’ transmigration [49], but also inhibits the adhesion between endothelial cells and vascular smooth muscle cells [50]. Indeed, there are no data showing that sEng directly inhibits the expression of membrane Eng. Thus, we propose that high sEng levels combined with aging inhibit Eng signaling in the aorta. The results of the study are summarized in Figure 6.

In conclusion, we demonstrated for the first time that long-term exposure to high levels of sEng during aging results in alteration of vasoconstriction properties of the aorta decreased Eng expression and signaling, and reduced eNOS phosphorylation. These findings suggest that sEng can be considered a risk factor for the development of vascular dysfunction during aging and a potential therapeutical target for pharmacological intervention.

## Figures and Tables

**Table 1:** List of primary and secondary antibodies used for Western blot analysis.

	Primary Antibody		Secondary Antibody	
Protein	Source	Dilution	Source	Dilution
Endoglin	(sc-19793) (Santa Cruz Biotechnology, Inc. Dallas, TX, USA	1:200	Sigma-Aldrich (A5420)	1:5000
eNOS	(sc-654) (Santa Cruz Biotechnology, Inc. Dallas, TX, USA	1:200	Abcam (ab6112)	1:2000
p-eNOS	(sc-21871-R) (Santa Cruz Biotechnology, Inc. Dallas, TX, USA	1:200	Abcam (ab6112)	1:2000
ID1	(ab134163), (Abcam, Cambridge, UK)	1:1000	Abcam (ab6112)	1:2000
VCAM-1	(32653S), (Cell Signaling Technology, Inc., Danvers, MA, USA)	1:1000	Abcam (ab6112)	1:2000
MLCK	(ab76092), (Abcam, Cambridge, UK)	1:200	Abcam (ab6112)	1:1000
p-MLCK	(44-1085G), (Invitrogen, Waltham, MA, USA)	1:1000	Abcam (ab6112)	1:2000
GAPDH	(G8795) (Sigma-Aldrich, St. Louis, MO, USA)	1:10,000	Sigma-Aldrich (A9917)	1:20,000

## Data Availability

All data collected in the manuscript are available upon request.
